# Receptors and Signaling Pathways for Recognition of Bacteria in Livestock and Crops: Prospects for Beneficial Microbes in Healthy Growth Strategies

**DOI:** 10.3389/fimmu.2018.02223

**Published:** 2018-09-27

**Authors:** Julio Villena, Haruki Kitazawa, Saskia C. M. Van Wees, Corné M. J. Pieterse, Hideki Takahashi

**Affiliations:** ^1^Laboratory of Immunobiotechnology, Reference Centre for Lactobacilli (CERELA-CONICET), Tucuman, Argentina; ^2^Food and Feed Immunology Group, Laboratory of Animal Products Chemistry, Graduate School of Agricultural Science, Tohoku University, Sendai, Japan; ^3^Livestock Immunology Unit, International Education and Research Center for Food Agricultural Immunology, Graduate School of Agricultural Science, Tohoku University, Sendai, Japan; ^4^Plant–Microbe Interactions, Department of Biology, Science4life, Utrecht University, Utrecht, Netherlands; ^5^Laboratory of Plant Pathology, Graduate School of Agricultural Science, Tohoku University, Sendai, Japan; ^6^Plant Immunology Unit, International Education and Research Center for Food Agricultural Immunology, Graduate School of Agricultural Science, Tohoku University, Sendai, Japan

**Keywords:** beneficial microbes, pattern recognition receptors, animal immunity, plant immunity, agricultural immunology

## Abstract

Modern animal and crop production practices are associated with the regular use of antimicrobials, potentially increasing selection pressure on bacteria to become resistant. Alternative approaches are needed in order to satisfy the demands of the growing human population without the indiscriminate use of antimicrobials. Researchers have brought a different perspective to solve this problem and have emphasized the exploitation of animal- and plant-associated microorganisms that are beneficial to their hosts through the modulation of the innate immune system. There is increasing evidence that plants and animals employ microbial perception and defense pathways that closely resemble each other. Formation of pattern recognition receptor (PRR) complexes involving leucine-rich repeat (LRR)-containing proteins, mitogen-activated protein kinase (MAPK)-mediated activation of immune response genes, and subsequent production of antimicrobial products and reactive oxygen species (ROS) and nitric oxide (NO) to improve defenses against pathogens, add to the list of similarities between both systems. Recent pioneering work has identified that animal and plant cells use similar receptors for sensing beneficial commensal microbes that are important for the maintenance of the host's health. Here, we reviewed the current knowledge about the molecular mechanisms involved in the recognition of pathogenic and commensal microbes by the innate immune systems of animal and plants highlighting their differences and similarities. In addition, we discuss the idea of using beneficial microbes to modulate animal and plant immune systems in order to improve the resistance to infections and reduce the use of antimicrobial compounds.

## The use of antimicrobials in livestock and crops: a global problem

The increasing prevalence of antimicrobial resistance in pathogenic microorganisms of clinical importance exerts a tremendous pressure on human healthcare systems globally. There is a dramatic rise in the prevalence of infections caused by multidrug- or extremely drug-resistant pathogens, which is estimated to cause several hundred thousand deaths annually (1, 2). Perhaps the best examples are the infections caused by multidrug-resistant bacteria belonging to the *Enterobacteriaceae* group. This fact implies a great concern since these pathogens are common natural inhabitants of human and animal microbiomes. Moreover, infections caused by this group of bacteria are often associated with prolonged hospitalization, elevated costs and high mortality rates (1, 2). Resistance to antimicrobials is a naturally occurring phenomenon. However, the increasing use of antimicrobials by the mankind has created a strong and unnaturally high selection pressure for resistant microorganisms. The emergence and spread of antibiotics resistance in microorganisms has been accelerated around the world by several human behaviors, including inappropriate use of antimicrobial substances, poor prevention of infectious diseases, defective control of infected patients within healthcare systems, and the insufficient control of antibiotics release into the environment.

Agricultural and food industries have been benefited from the availability of antimicrobial compounds for animal production and crop protection. Antibiotics have been widely used in livestock diets during the past several decades due to their therapeutic effects (3, 4). Antibiotics are able to reduce the frequency of diarrhea and improve performance parameters like body weight gain or feed conversion ratio. These beneficial effects of feed antibiotics are generally explained by modifications of the intestinal bacteria and their interaction with the animal host, including bacterial interactions with intestinal tissues and the immune system (3, 4). Another class of agents used in agriculture are cationic metals that can be included in animal diets as nutritional supplements or spread on pastures to support crop growth and protection. Heavy metals, in particular, give rise to concerns among public health professionals, as they can persist in the environment for prolonged periods. Moreover, bacteria can also exhibit resistance to these chemical elements and the genes encoding this phenotype can be physically localized on plasmids that may also contain one or more antimicrobial resistance-encoding genes (5).

On the other hand, chemical pesticides including antimicrobials for protection of crops against bacterial plant diseases are limited in availability, use, and efficacy, and their affectivity is limited. Such antimicrobials are used for managing bacterial plant diseases of fruit trees, for which it has been proven to be economically feasible (6). Although the amount of antibacterial antibiotics used on plants is small compared to medical, veterinary, and livestock production uses, antibiotics-resistant bacterial plant pathogens have emerged, which further complicates the control of bacterial diseases of crops, especially fruit trees. In addition, the pollution with antimicrobial, mutagenic and carcinogenic compounds in aquatic and soil environments caused by the discharge industrial wastes, atmospheric deposition, and fertilizers is an emerging public health concern because of the potential in producing drug-resistant microbes that can be up-taken by food crops (7).

Antibiotics-resistant microorganisms of agricultural origin have significant public health implications since they can be transmitted to humans through the environment (8), and food products (9), and to agricultural workers by direct contact (10). It was suggested that repeated exposure to low doses of antimicrobial agents, that is the context in which growth-promoting antimicrobials are administered, creates ideal conditions for the emergence and spread of antibiotics-resistant bacteria (11).

Because of the concern that the use of antimicrobials in agricultural industry might contribute to a rise in bacterial antibiotics resistance, the use of some types of antibiotics have been restricted by some countries since the 1970's. In this regard, the European Union introduced a total ban on the application of antibiotics as feed additives from 2006 onwards (3, 12). These regulatory issues about the ban of antibiotic growth promoters together with the consumer's demand for a safe food production system have stimulated the search for alternative strategies to improve resistance against pathogens, promote growth and health of livestock (13, 14), and minimize the impact of the industry on the environment (3, 4).

In order to control infections, scientists poned that the modulation of animal and plant immune systems by using beneficial microbes able to confer health-promoting activities would be an interesting alternative. Plant and animal innate immune systems respond to pathogen infections but also regulate beneficial interactions with commensal and symbiotic microbes (15). Recent pioneering work revealed striking similarities between the molecular organization of animal and plant systems for non-self-recognition and anti-microbial defense. Studies have also identified that animal and plant cells use similar receptors for sensing beneficial commensal microbes that are important for the maintenance of the host's health. In this review we highlight current knowledge on the molecular mechanisms involved in the recognition of pathogenic and commensal microbes by the innate immune systems of animal and plants highlighting their differences and similarities. In addition, we discuss the idea of using beneficial microbes to modulate animal and plant immune systems to improve resistance to pathogen infections and to reduce the use of antimicrobial compounds in a biological way. The progress in our understanding of the cellular and molecular mechanisms of beneficial microbes-host interaction is reviewed in order to give a scientific basis for the design of new intervention strategies that can improve immune fitness of animals and plants in a more sustainable way.

## Innate immunity in animals and plants

Animals and plants have acquired the ability to recognize conserved microbial molecules that are characteristic of microorganisms but are not found in animal or plant hosts. The recognition of these microbial molecules is a key step in innate immune defenses, and is mediated by a set of receptors referred to as pattern-recognition receptors (PRRs) that recognize the microbe-associated molecules (16). “Pathogen-associated molecular patterns” (PAMPs) is the term generally used when referring to the molecules that elicit innate immune responses. As classically defined, PAMPs are evolutionarily conserved pathogen-derived molecules that distinguish hosts from pathogens. PAMPs include, among others, lipopolysaccharide (LPS), peptidoglycan, bacterial flagellin, and yeast mannans (17). However, because non-pathogenic microbes also possess such molecules, the term “pathogen-associated” is a misnomer and a more precise term would seem to be “microbe-associated molecular patterns” (MAMPs) (16). Therefore, to avoid confusion here, the term “MAMP” is used instead of “PAMP.”

**Figure 1:**
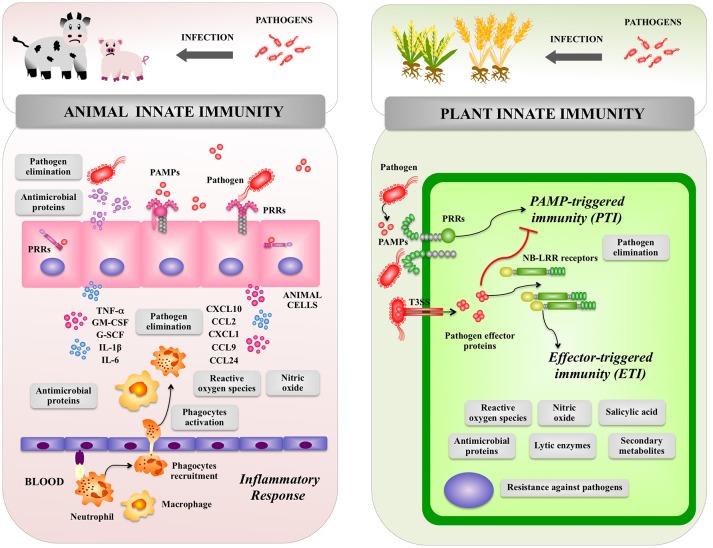
Global overview of animal and plant innate immune systems. The inflammatory response triggered by pathogens in the animal host as well as pattern recognition receptor-triggered immunity and effector triggered immunity triggered by pathogens in the plant host are shown. Pattern recognition receptor (PRR), pathogen-associated molecular patterns (PAMPs), type III secretion system (T3SS), PAMP triggered immunity (PTI), effector-triggered immunity (ETI), tumor necrosis factor alpha (TNF-α), interleukin (IL), chemokine (C–X–C motif) ligand (CXCL), C-C motif chemokine ligand (CCL), granulocyte-colony stimulating factor (G-CSF), Granulocyte-macrophage colony-stimulating factor (GM-CSF).

**Figure 2:**
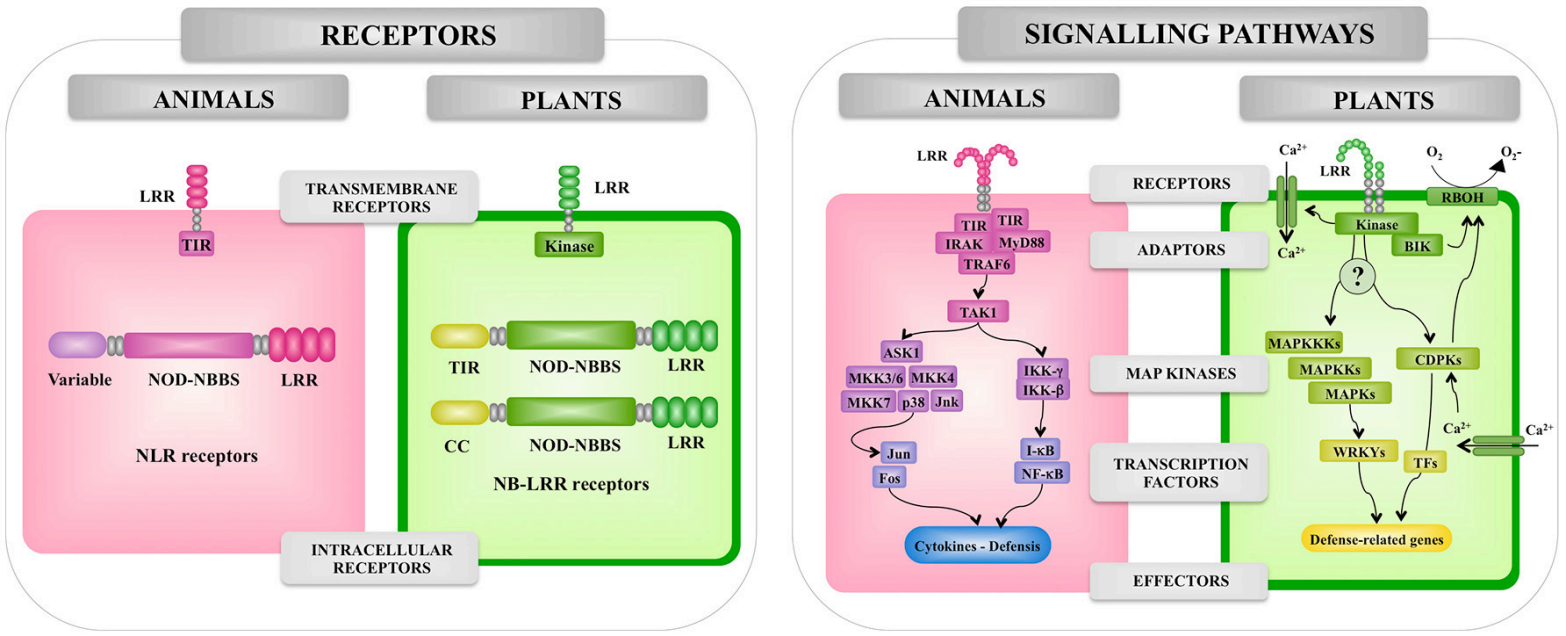
Comparison of the pattern recognition receptors and signaling pathways involved in the recognition of pathogenic microorganisms by animal and plant cells. Receptors, adaptors, signaling pathways, and effectors involved in the response of animal and plant cells to microbes are shown. Leucine-rich repeat (LRR), Toll/interleukin-1 receptor (TIR), nucleotide-binding oligomerization domain (NOD), myeloid differentiation primary response 88 (MyD88), TNF receptor associated factor (TRAF), interleukin-1 receptor-associated kinase (IRAK), mitogen-activated protein kinase (MAPK), (MAPKK), mitogen-activated protein kinase (MAPKKK), WRKY transcription factor (WRKY), nuclear factor kappa B (NF-kB), calcium-dependent protein kinase (CDPK), transcription factor (TF). NADPH oxidases are designated as RBOH in plants.

Remarkable similarities have been uncovered in the molecular mode of MAMP perception in animals and plants, including the discovery of plant receptors resembling mammalian Toll-like receptors (TLRs) or cytoplasmic nucleotide binding domain (NBD) and leucine-rich repeat (LRR) superfamily proteins (NLR) (15, 18). Changes in cytoplasmic Ca^2+^ levels, the production of reactive oxygen species (ROS) and nitric oxide (NO) as well as the post-translational activation of mitogen-activated protein kinase (MAPK) cascades are commonly reported to signal the activation of innate immune responses in plants (19). Intriguingly, most of these components have also been described to be of central importance to MAMP-induced activation of innate immune responses in animal cells (20). In addition, both plants and animals synthesize a wide range of small antimicrobial peptides and both produce an oxidative burst via conserved gp91phox NADPH oxidases after the pathogen encounter (16). Therefore, common features of innate immunity in animals and plants include defined receptors for microbe-associated molecules, conserved MAPK signaling cascades and the production of antimicrobial peptides and oxidative compounds (Figures 1, 2).

### Animal innate immune system

In animals, epithelial cells from the skin and mucosal surfaces, including those lining the respiratory, urogenital and gastrointestinal tracts, provide a physicochemical barrier between the host cells and the outside world including microorganisms. The mucosal surfaces and the skin also have an intricate network of immune cells that perform surveillance functions and have the ability to trigger defense mechanisms against invading pathogens. Antigen presenting cells including dendritic cells and macrophages reside in tissues throughout the body and are especially abundant in areas where infections are likely to arise. In addition, epithelial cells from skin and mucosal surfaces have also immune functions since they are able to deliver signals to immune cells when potentially dangerous microorganisms have reached the host (21, 22). Therefore, when a pathogen invades a tissue, epithelial and immune cells elicit an inflammatory response in order to limit its replication and dissemination (Figure 1). This response is characterized by an initial recognition of MAMPs of pathogens by PRRs expressed in epithelial and immune cells leading to production of immune factors including type I interferons (IFNs), cytokines and chemokines. Those biological mediators are responsible for the recruitment and activation of additional immune cells that participate in the innate immune response (21, 22). Changes in the microenvironment of infected tissue induce the blood vessels to dilate and become permeable to fluid and proteins. At the same time, the endothelial cells lining the local blood vessels are stimulated to express cell adhesion proteins that facilitate the attachment and extravasion of immune cells including neutrophils and monocytes (Figure 1). Both types of phagocytes possess an extraordinary capacity to kill pathogens through a wide range of antimicrobial agents including antimicrobial proteins and oxidative compounds. Pathogenic stimuli activate pathways in neutrophils and macrophages that signal for the phosphorylation and assembly of the NADPH oxidase that then produces superoxide and H_2_O_2_ in a process known as the respiratory burst. In addition, the inducible enzyme NO synthase (iNOS) is expressed in phagocytes leading to NO production that is a gas with highly reactive properties (21, 22).

### Plant innate immune system

The entry of pathogenic bacteria into the plants' tissues is the first and most important step in infectious diseases. Foliar bacterial pathogens mainly enter into the plant cells through the open stomata, water pores, or physical injuries (23, 24). Bacteria colonize the plant apoplast of a leaf, extensively multiply in the apoplast and inject several immune-suppressive effector molecules into plant cells through the type III secretion system (TTSS) (Figure 1). Those effector molecules induce visible disease-associated necrosis and chlorosis (25). On the other hand, bacterial pathogens living in the rhizosphere invade root tissues through small wounds after which they colonize intercellular spaces and stem tissues (26). Then, plants develop symptoms, such as bacterial wilt that are caused by suppressed water fluxes (26), or damping-off and root rot symptoms caused by degradation enzymes and toxins secreted by bacteria in vascular tissues (27).

In order to protect themselves against bacterial pathogens, plants have developed highly effective defense systems. In plants, there are no specialized immune cells such as macrophages, neutrophils, or dendritic cells that are the key players of the animal immune system. In contrast, plants are autonomously capable of recognizing the presence of pathogens and trigger defense responses at the level of each single cell (Figure 1). As plants are lacking in mobile immune cells and the cellular adaptive immune systems, they are mainly dependent on innate immunity for protection against pathogens including efficient signaling mechanisms, which is now so-called the plant immune system (15).

Once the pathogen breaks the primary defense barriers, e.g., the stomata-mediated defense system, plants can detect several MAMPs including flagellin, translational elongation factor Tu (EF-Tu), cold-shock protein (CSP) or LPS (27–29). Recognition of MAMPs by PRRs locating at the plasma membrane of plant cells activates downstream signaling cascade and a series of defense responses including the synthesis of phytoalexins, cell wall strengthening, and accumulation of pathogenesis-related proteins such as lytic enzymes (chitinases, glucanases, and proteases) (Figure 1) (30–32). The MAMP-triggered immune system, which is named “PAMP triggered immunity” (PTI), prevents the establishment of infection in non-host plants.

Bacterial pathogens have various virulence strategies that inactivate PTI. For example, toxins produced by pathogenic bacteria are able to change plant metabolism in order to establish an advantageous environment for bacterial colonization. In addition, several bacterial pathogens have developed strategies to evade PTI. Multiple effector molecules are delivered by bacterial pathogens into plant cells through the TTSS in order to suppress PTI at various steps of the defense signaling pathways that confer disease resistance (33).

Plants have a second class of immune receptors that include intracellular immune receptors called resistance proteins (NB-LRR receptors). These intracellular receptors directly or indirectly recognize effectors secreted by pathogens into the host intracellular environment and activate effector-triggered immunity (ETI), which is often associated with rapid cell death, production of ROS and salicylic acid (SA), and the expression of defense-related genes (Figure 1) (34). Activation of ETI enables plants to respond rapidly and efficiently to virulent pathogens (35). In PTI and ETI, the production of ROS is an important early defense mechanism as in the innate immune response of animals (26, 33). Extracellular generation of ROS during the oxidative burst of plants depends on transient increases of cytosolic Ca^2+^ levels and appears to be mechanistically similar to the respiratory burst of animal phagocytes, which is catalyzed by an NADPH oxidase protein complex. Plants harbor a family of genes with significant homology to the human gene encoding the catalytic subunit gp91 of the NADPH oxidase complex. In addition, NO was found to be produced upon treatment of plants with MAMPs as well as upon pathogen infection, suggesting that it may be important for the activation of innate defense mechanisms (36).

## Receptors for bacterial recognition in animals and plants

The ability to distinguish between self and non-self-antigens is an important feature of all living organisms and forms the basis for the activation of innate defense mechanisms against infections (15). In animals and plants, innate immunity involves both cell surface receptors (19) and intracellular receptors of the NLR superfamily (37) (Figure 2).

### Pattern recognition receptors for bacteria in animals

As mentioned before, microbial recognition by the innate immune system of animals occurs via a range of germline-encoded PRRs such as the TLR family, the NLR family, the RIG-I-like RNA helicases, the C-type lectin receptors, and cytosolic DNA sensors (38, 39). The interaction of microbial ligands with PRRs induce the activation of the innate immune system leading to diverse cellular responses including the induction of interferon regulatory factors (IRFs), activator protein-1 (AP-1), and nuclear factor-kappa B (NF-κB) that regulate the expression of cytokines, chemokines, and type I IFNs.

TLRs were the first PRRs discovered and they are the best-characterized family of PRRs. Initially, the Toll pathway was described in the pattern formation in early drosophila embryo development (40). Later, the cytoplasmic domain of Toll (the Toll–interleukin 1 (IL-1) receptor (TIR) domain) was found to have homology with the cytoplasmic domain of human IL-1 (41). In addition, the study of antimicrobial peptides genes in drosophila and their promoters suggested that they were regulated by NF-κB-like transcription factors that also function in the Toll pathway (42). Meanwhile, a human homolog of Toll was shown to activate expression of NF-κB controlled genes (43), and a year later TLR4 was identified as the LPS sensor (44).

**Figure 3:**
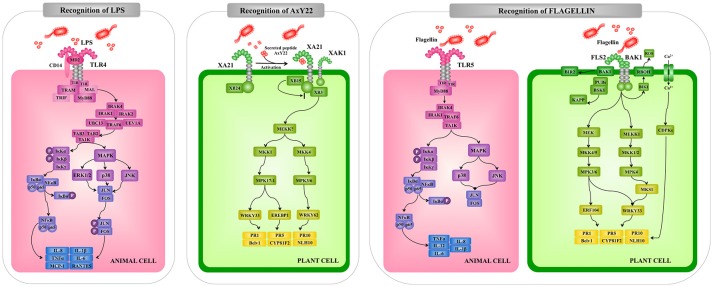
Comparison of the extracellular pattern recognition receptors and signaling pathways involved in the recognition of pathogenic microorganisms by animal and plant cells. Recognition and signaling pathway mediated by animal Toll-like receptor (TLR)-4 and TLR5 and plant receptors XA21 and FLS2 are shown as examples of extracellular microbial recognition. Toll/interleukin-1 receptor (TIR), myeloid differentiation primary response 88 (MyD88), TNF receptor associated factor (TRAF), interleukin-1 receptor-associated kinase (IRAK), mitogen-activated protein kinase (MAPK), mitogen-activated protein kinase (MKK), nuclear factor kappa B (NF-kB), tumor necrosis factor alpha (TNF-α), interleukin (IL), *Arabidopsis thaliana* receptor kinase FLS2 (FLS2), rice receptor kinase XA21 (XA21), reactive oxygen species (ROS), calcium-dependent protein kinase (CDPK), pathogenesis-related protein (PR).

TLRs are characterized by an extracellular LRR domain and an intracellular TIR protein-protein interaction domain. TLRs are coupled to signaling adaptors such as MyD88, which also have TIR domains. Activation of the TLR signaling cascade results in the nuclear translocation of NF-κB-like transcription factors, leading to the production of antimicrobial peptides in both insects and vertebrates and signaling molecules such as cytokines and chemokines in vertebrates (16) (Figure 3).

Upon interaction with their ligands, TLRs dimerize and initiate two signaling pathways: the MyD88-dependent and MyD88-independent pathways. These two types of cellular responses are mediated by a selective use of adaptor molecules recruited to the TIR domains. Four adaptor molecules have been identified so far: MyD88, TIR-associated protein (TIRAP), TIR domain-containing adaptor protein-inducing IFN-β (TRIF), and TRIF-related adaptor molecules (TRAM). MyD88 and TIRAP are responsible for the induction of pro-inflammatory genes, and TRIF and TRAM induce IFNs (45, 46).

All TLRs, except TLR3, signal through MyD88. In MyD88-dependent signaling, MyD88 is recruited to and associates with the cytoplasmic domain of the TLRs via interaction with the TIR domains (Figure 3). Then IL-1R-associated kinase 1 (IRAK-1) and IRAK-4 are recruited and activated by phosphorylation. Activated IRAK-4 phosphorylates IRAK-1, which subsequently associates with Tumor necrosis factor receptor (TNFR)-associated factor 6 (TRAF6). TRAF6 activates transforming growth factor (TGF)-activating kinase 1 (TAK1) and this factor then phosphorylates IKK-b and MAPK kinase 6 (MKK6), leading to degradation of I-kB and NF-kB nuclear translocation. The final response is the induction of genes involved in innate defense mechanisms. Activation of the MyD88-dependent pathway also results in the activation of MAPK-p38, MAPK-ERK, and MAPK-JNK, which leads to the activation of AP-1 (45–47).

**Figure 4:**
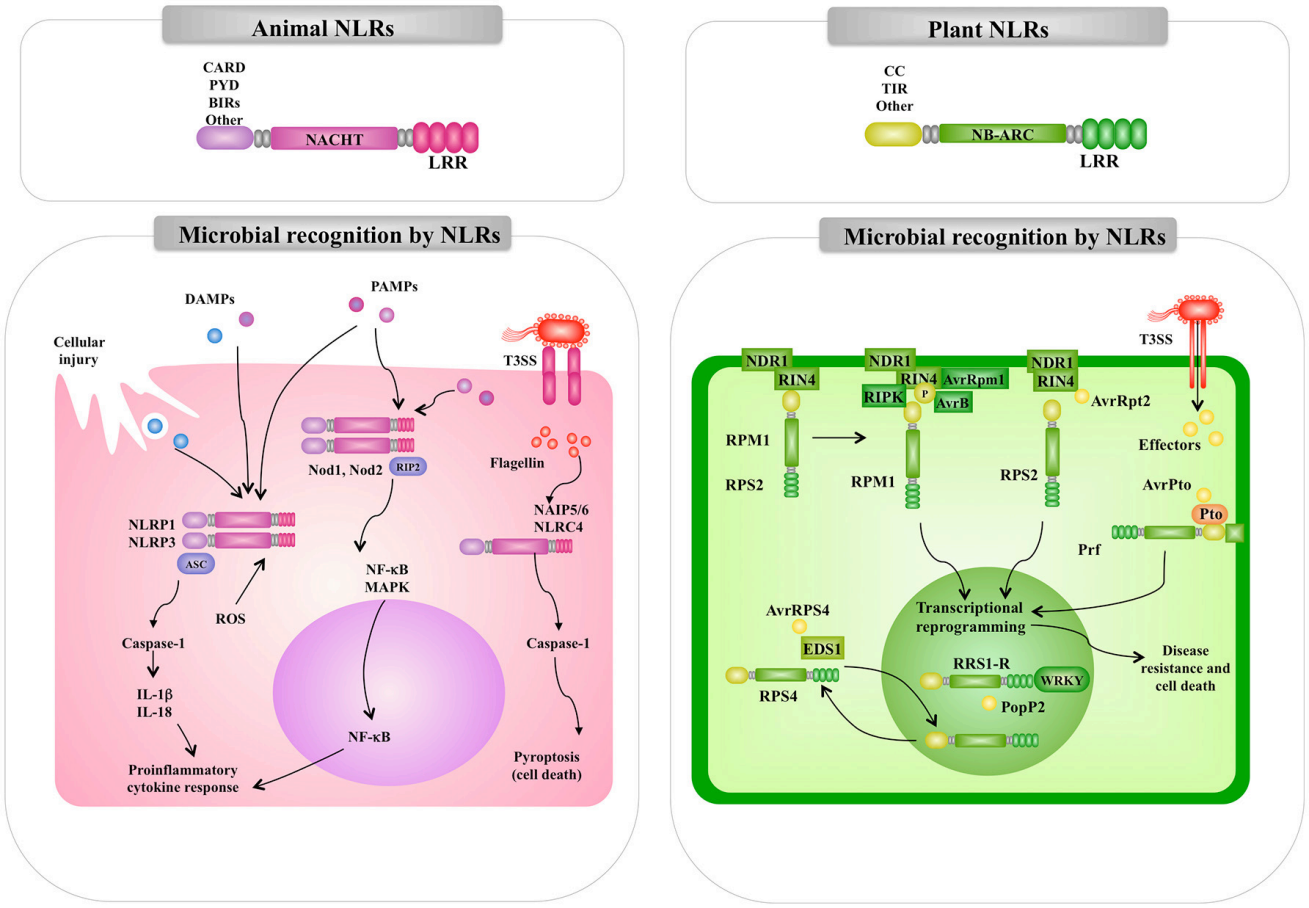
Comparison of the intracellular pattern recognition receptors and signaling pathways involved in the recognition of pathogenic microorganisms by animal and plant cells. Recognition and signaling pathway mediated by animal and plant nucleotide binding domain (NBD) and leucine-rich repeat (LRR) superfamily proteins (NLR) are shown as examples of intracellular microbial recognition. Caspase-activated recruitment domains (CARD), central nucleotide-binding (NACHT), pathogen-associated molecular patterns (PAMPs), microbe-associated molecular patterns (MAMPs), danger-associated molecular patterns (DAMPs), mitogen-activated protein kinase (MAPK), nuclear factor kappa B (NF-kB), interleukin (IL), NLR family pyrin domain containing (NLPR), reactive oxygen species (ROS).

In addition to the transmembrane TLRs, mammals have a family of cytosolic PRRs that belong to a family of proteins referred to as NLR proteins that are involved in apoptotic and inflammatory responses (48). NLR proteins are characterized by a tripartite domain architecture consisting of a variable N-terminal domain, a central nucleotide-binding domain and C-terminal LRRs (Figure 4).

The NOD1 and NOD2 receptors were the first members of the NLR family to be reported as intracellular sensors for microorganisms in mammals. The interaction of NLRs with their ligands triggers signaling cascades that induce the translocation of NF-kB, and the production of cytokines and chemokines (49). NOD1 and NOD2 receptors are located in the cytosol of host cells. However, after their interaction with their ligands they undergo redistribution to the plasma membrane from where they initiate signaling pathways (50). It should be mentioned that differences in the expression of the two receptors exists. NOD2 expression is limited to cells of hematopoietic origin and some types of epithelial cells (specially the gastrointestinal mucosa) while NOD1 is ubiquitously expressed in most cell types (51). Interestingly, the expression of NOD1 and NOD2 in macrophages can be increased by stimulation with LPS or proinflammatory cytokines such as tumor necrosis factor (TNF)-α, interleukin (IL)-1β, and IL-6 (52). Both, NOD1 and NOD2 have three domains: a C-terminal domain containing LRRs, a central nucleotide-binding (NACHT) domain, and a N-terminal effector-binding domain composed of caspase-activated recruitment domains (CARD) (53). The C-terminal domain is responsible for ligand interaction while NACHT domain allows self-oligomerization and is necessary for self-activation. NOD1 has one CARD domain while NOD2 has two series-wound CARDs that mediate the interaction with intracellular proteins that form the signaling platform (53). Similar to TLRs, the major outcome of NOD1 and NOD2 signaling pathways are the activation of NF-κB, MAPK-p38, MAPK-JNK, and MAPK-ERK, with the subsequent production of inflammatory factors (54) (Figure 4). Signaling through NOD pathways involves the recruitment of the receptor-interacting protein 2 (RIP2). The adaptor protein RIP2 is a serine/threonine protein kinase that possesses a C-terminal CARD domain that allows its interaction with NOD1 and NOD2 (55). The kinase activity of RIP2 is regulated by ubiquitination and phosphorylation. Ubiquitination of RIP2 induces the recruitment of TAK1 and the subsequent recruitment of IKK kinase complexes (IKK-α, IKK-β, and IKK-γ) leading to phosphorylation and degradation of IκBα (56).

Animal NLR proteins also participate in the formation of inflammasomes (Figure 4). The inflammasomes are multiprotein platforms with cytosolic sensors for a wide range of MAMPs or damage-associated molecular patterns (DAMPs) (57, 58). Inflammasomes participate in the defense against bacterial pathogens through the activation of inflammatory caspases. As mentioned before, flagellin can be recognized by the membrane expressed-TLR5. However, this MAMP can also be delivered into the cytosol by the secretion systems present in pathogenic bacteria such as the type III (T3SS) and type IV (T4SS) secretion systems present in *Salmonella typhimurium* or *Legionella pneumophila*, respectively. Studies have established that the neuronal apoptosis inhibitory protein (NAIP)-NLRC4 inflammasome plays a critical role in anti-bacteria defenses (58). In the NAIP-NLRC4 inflammasome, NAIPs are the cytosolic receptors for flagellin and secretion systems proteins while the NLRC4 is the adapter for caspase-1 activation (57). It has been established that component of flagella are sensed by NAIP5 and NAIP6, whereas components from bacterial secretion systems are detected by NAIP1 and NAIP2 (57). Inflammasome-dependent caspase-1 activation participates in the maturation and secretion of the inflammatory factors IL-1β and IL-18, and triggers a proinflammatory form of cell death referred as to pyroptosis. Several other animal NLR proteins are involved in inflammasomes formation and innate immune responses against bacterial pathogens [for a review see (59)].

### Pattern recognition receptors for bacteria in plants

Plant cells encounter a variety of microbial-associated signals when interacting with microorganisms *in vivo*, and the plant's ability to recognize complex MAMPs is likely to determine its efficiency in inducing innate defense mechanisms. Various pathogenic Gram-negative bacteria harbor LPS and flagellin, which stimulate plant defenses (60). These MAMPs bind to PRRs and trigger the expression of immune response genes and the production of antimicrobial compounds (61).

*Arabidopsis* FLS2 and EFR, which can recognize a conserved 22-amino acid peptide sequence in bacterial flagellin and active epitope of bacterial elongation factor (EF)-Tu, respectively, are typical LRR-receptor-like kinases (RLKs) acting as PRRs (62, 63). Rice receptor kinase XA21 has been identified as a rice resistance gene product conferring race-specific resistance to *Xanthomonas oryzae* pv *oryzae* (64). It has been shown to perceive ax21 protein which seems to play a role in quorum sensing (65). Interestingly, the plant RLKs have structural and functional similarity to animal TLR proteins that can recognize MAMPs of bacterial pathogens in animals thereby inducing innate immunity (18). For example, FLS2 and TLR5 are equally perceptive to bacterial flagellin, suggesting that both PRRs are conserved by convergent evolution. Interestingly, bacterial *Pseudomonas* pathogens have been shown to evade animal and plant immunity through the activity of the protease AprA, which degrades flagellin monomers, therewith escaping detection by the host's immune system (66). On the other hand, the downstream signaling pathways activated by recognition of MAMPs through PRRs are diversified between plants and animals, while the production of ROS, transient increases of cytosolic Ca^2+^ levels followed by activation of calcium-dependent protein kinase (CDPKs), activation of MAPK cascades and NO-mediated signaling seem to be common signaling events (Figure 1).

The best-characterized plant immune receptors are a large class of intracellular receptors often called NBS-LRR pathogen-resistance proteins, which have an overall tripartite structure similar to that of the mammalian NLR proteins (67). In general, plants have large families of these NLR proteins; *Arabidopsis* has 140 (68) and rice has over 500 (69). Most of the NLR pathogen-resistance proteins have either a TIR or a coiled-coil (CC) N-terminal domain (Figure 4). In contrast to the animal NOD1, NOD2, and NALP3 proteins, which respond to peptidoglycan degradation products, the plant NLR pathogen-resistance proteins directly or indirectly recognize pathogen effector molecules thereby activating downstream signaling pathways for induction of defense response.

It should be noted that a differential aspect between plant immune system and innate immune system in animals is that PRRs and NOD receptors equally contribute to recognize PAMPs/MAMPs in animals (Figures 3, 4). However, the plant immune system consists of two-layered defense system: first PTI is mediated through recognition of MAMPs by PRRs and second ETI is mediated through recognition of pathogen effector molecules by NB-LRR receptors (Figure 1).

## Modulation of the immune system by beneficial microbes in animals and plants

Today, the world faces the enormous challenge of improving the production of livestock and crops without the indiscriminate use of antimicrobials (70). Thus, alternative approaches are needed in order to satisfy the demands of the growing human population. Scientists have brought a different perspective to solve this problem and have emphasize on the exploitation of animal- and plant-associated microorganisms that are beneficial to their hosts through the modulation of the innate immune system.

### Beneficial microbes for animals

**Figure 5:**
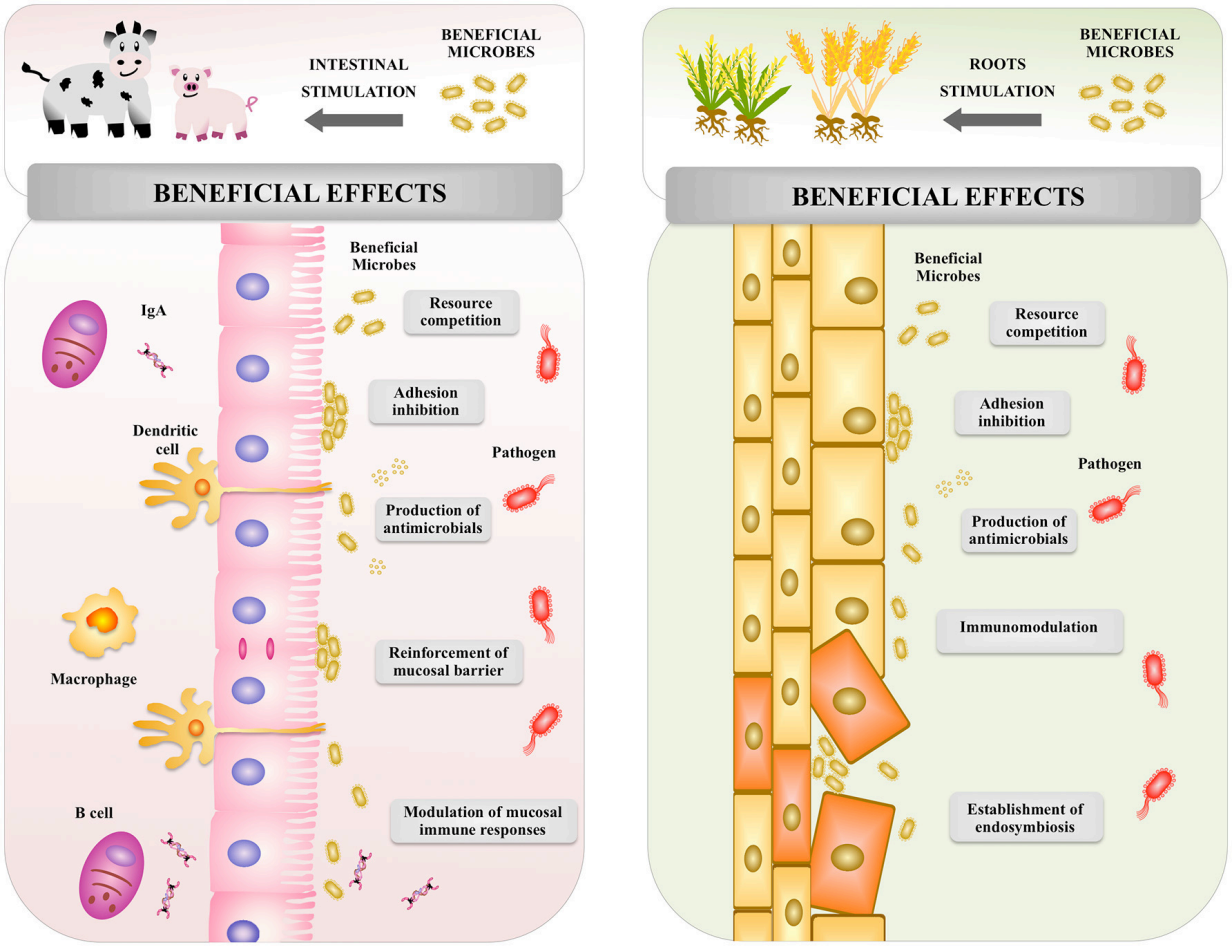
Global overview of the effect of beneficial microbes on animal and plant hosts. Beneficial microbes in both animal and plants are able to increase resistance to pathogenic microorganisms by using similar mechanisms: competition for nutrients, inhibition of adhesion to host's cells, production of antimicrobial molecules, and modulation of host's immune system.

Studies in humans and animals have shown that beneficial microbes in the gut are able to confer several health benefits (Figure 5), including the stimulation of intestinal epithelial cell proliferation, the reinforcement and maintenance of tight junctions, the expression of antimicrobial factors, and the modulation of the mucosal immune system (46, 71). It has been demonstrated that for most of these beneficial effects PRRs play a key role in the interaction of microbes with host cells (46, 71, 72).

It was reported that commensal bacteria, through the activation of TLR2, modulate the organization of tight junctions proteins (73), improve transepithelial resistance (74), and induce a rapid reshaping and stretching of epithelial cells after injury (75). In addition, activation of TLR2 and TLR4 in the animal gut have been shown to be involved in the expression of trefoil factor 3 (TFF3), epidermal growth factor receptor (EGFR), amphiregulin, and prostaglandin E2, which are important factors in wound healing and repair of the intestinal mucosa (75, 76). These studies demonstrated an important role of animal beneficial microbes in the maintenance of intestinal barrier function.

Antimicrobial peptides are constitutively produced in the animal gastrointestinal tract. However, their expression can be improved by commensal MAMPs through the activation of TLRs or NLRs (77). Antimicrobial compounds, such as regenerating islet-derived 3 (Reg3)-β protein, Reg3-γ, CRP-ductin, resistin-like molecule-β, and β-defensins are induced in intestinal epithelial cells and Paneth cells by microbial products through MyD88-dependent signaling (78–80). Interestingly, it was recently reported that the metabolic activity of intestinal microbiota also influences the production of antimicrobial factors (81). The study demonstrated that short-chain fatty acids produced by microbiota promoted the production of Reg3-γ and β-defensins in intestinal epithelial cells. The effect of short-chain fatty acids were dependent on G protein-coupled receptor (GPR), mammalian target of rapamycin (mTOR), and signal transducer and activator of transcription protein (STAT)-3 signaling. The data thereby provided a novel pathway by which animal beneficial microbes modulate the expression of antimicrobial compounds in the gut (81).

On the other hand, a plethora of evidence supports the active role of commensal bacteria in the development and maintenance of intestinal immune homeostasis in the animal host (21, 82, 83). The molecular communication between microbes and intestinal epithelial cells, and the role of this interaction in the promotion of immune homeostasis have been subjects of intense research (21). PRR signal transduction triggered by pathogens induce proinflammatory responses by intestinal epithelial cells that significantly influence the behavior of the underlying lamina propria immune cells. This PRR signaling is crucial to protect the animal hosts against infections (80, 84). In addition, intestinal epithelial cells are able to sense commensal and beneficial microbes that help to maintain immune status and inhibit excessive inflammation. Products derived from animal intestinal microbiota activate TLRs in intestinal epithelial cells, and increase the expression of negative regulators of the TLRs signaling pathway such as IRAK-M, TOLLIP, SIGIRR, A20, and peroxisome proliferator-activated receptor-γ (PPARγ). Through this mechanism, animal beneficial microbes help to control intestinal inflammatory responses (85, 86). Microbial products also stimulate the expression of proliferation-inducing ligand (APRIL) and B cell-activating factor (BAFF) (87). Both, APRIL and BAFF promote IgA class-switching responses in the intestine and are involved in the maintenance of the appropriate levels of secretory IgA antibodies that protect mucosal surfaces. In addition, interactions of animal beneficial microbes with intestinal epithelial cells modulate the function of antigen presenting cells. Intestinal dendritic cells are influenced by factors produced by the intestinal epithelium including transforming growth factor (TGF)-β, retinoic acid and thymic stromal lymphopoietin to acquire a tolerogenic phenotype (88).

Scientists have isolated and select specific microbial strains in order to improve immune functions in human and animals. The strains used to improve the health of human and animals through the modulation of the immune system are referred to as immunomodulatory probiotics or immunobiotics (46, 71, 72, 89, 90). Studies from the last decades have shown that immunobiotics are able to beneficially modulate the intestinal immune system of human (90, 91), porcine (89, 90, 92), and bovine (46, 72) hosts. Immunobiotics allow an efficient control of inflammatory responses in the gut and an improved protection against infectious diseases.

In addition, researchers have demonstrated the immunomodulatory beneficial effect of commensal microbes and immunobiotics can be extended beyond the intestinal tract. It was reported that orally administered immunobiotics are able to differentially modulate immune responses in distal mucosal tissues such as the respiratory tract (93, 94) or mammary glands (95). The release of microbial immunomodulatory molecules in the intestine that are transported to distal sites (11, 96, 97); the mobilization of immune cells from the gut into the blood and distal tissues (98–100), and the systemic metabolic reprogramming that induce the production of immunomodulatory metabolites (101) have been proposed to explain the effects of gut animal beneficial microbes on systemic and distal mucosal tissue responses.

### Beneficial microbes for plants

The interaction of plants with particular microorganisms can be beneficial (Figure 5). Microbial populations that influence the growth, development, and health of plants can be found below and above the ground, as well as within the plant (102). Commensal and mutualistic microbes are able to provide plants essential benefits including nitrogen fixation, enhanced mineral uptake, and growth promotion (103). Microbes also help plants to resist a variety of stresses, including toxins, heavy metals, drought, salinity, and extreme temperature (70). Of interest, plants and microbes interaction modulates the plant innate immune system and improves protection from pathogens (104–106).

*Pseudomonas simiae* WCS417r (formerly known as *Pseudomonas fluorescens* WCS417r) is a non-pathogenic rhizobacterial strain that colonizes the rhizosphere in the regions where plants produce exudates and lysates (107). This strain is capable to suppress soil-borne diseases caused by infection with a broad range of pathogens (108). Beneficial rhizobacteria strains have the potential to reduce the incidence of infectious diseases through the activation of a plant-mediated defense system termed “induced systemic resistance” (ISR) (109). *Arabidopsis* activation of ISR by treatment of the roots with *P. simiae* WCS417r is not accompanied by salicylic acid (SA)-responsive PR-protein gene expression, indicating that WCS417r-mediated ISR functions independently of this plant hormone (110). This is in contrast to findings in other plant species including rice, tobacco, cucumber and tomato where SA-independent ISR has been demonstrated (11, 111, 112). Indeed, a series of studies on signaling pathways required for *P. simiae* WCS417r-mediated ISR using *Arabidopsis* mutants indicated that jasmonic acid (JA)- and ethylene (ET)-dependent signaling play a central role in the regulation of ISR (113–115). Indeed, ET accumulation is a well-known response to MAMP recognition (28). Furthermore, transcriptional co-activator NPR1 and the root-specific transcriptional regulator MYB72 have also been implicated in JA/ET-dependent ISR by *P. simiae* WCS417r (113, 116, 117). Recently, it was shown that MYB72 plays an important role in the rhizobacteria-induced excretion of antimicrobial coumarins that shape the assembly of the microbiome in the rhizosphere, potentially to optimize associations with ISR-inducing rhizobacteria (118).

While colonization of the roots by rhizobacteria is not generally accompanied with up- or down-regulation of defense-related gene expression or increases in the production of JA and ET, plants have enhanced defensive capacity to a broad-range of pathogens. This enhanced ability to induce basal defense system is termed “priming” (119, 120). Global gene expression analysis of *P. simiae* WCS417r-mediated ISR revealed that JA- and/or ET-responsive defense-related gene were primed for enhanced expression in response to challenge infection by the phytopathogenic bacteria *P. syringae* (121–123). In *Arabidopsis*, the levels of transcription factors of the AP2/ERF family including MYC2 are especially increased in the ISR-primed state (124). Since rhizobacteria-mediated ISR was compromised in MYC2-muagenized *jin1 Arabidopsis* mutants (125), MYC2 seems to play a key role to regulate priming during ISR. Furthermore, epigenetic regulation of defense-related gene expression including DNA methylation and chromatin re-modulation seems to be associated with the priming phenomenon (126, 127). However, the relation of rhizobacteria colonization and epigenetic regulator mechanism remains to be investigated.

Other beneficial non-pathogenic microorganisms including bacteria, fungi, and oomycetes including *Bacillus* spp. *Serratia liquefaciens, Penicillum* spp. *Phoma* spp. *Trichderma* spp. and *Pythium oligandrum* also induce systemic resistance including ISR to a broad range of pathogens through activation of SA, JA, or ET-responsive defense-related genes in plants. (128–133). In this regard, when tomato roots are treated with a mycelial homogenate of the non-pathogenic oomycete *P. oligandrum*, bacterial wilt disease caused by *Ralstonia solanacearum* is suppressed (134). In addition, the treatment of tomato root cells with *P. oligandrum* elicitin also induced JA/ET-responsive defense-related gene expression and inhibited the occurrence of bacterial wilt disease (132–134). Moreover, this response can be also obtained in *jai1* mutants in which JA-signaling pathway is impaired (112). Thus, the elicitin of *P. oligandrum* seems to be recognized as MAMP by tomato cells thereby activating PTI. Hence, activation of defense system mediated by recognizing MAMPs of beneficial microorganisms seems to contribute their disease suppressive activity.

Recently, it was reported that insect pathogens colonizing the surface of plant leaves or natural soils have the potential to activate the plant defense system (135). *Bacillus thuringiensis* is a well-known pathogen that causes disease in caterpillars of various types of moths and butterflies by producing δ-endotoxins. The treatment of tomato roots with cell-free culture filtrate medium of *B. thuringiensis* suppressed bacterial wilt disease caused by *R. solanacearum* with systemic induction of ET-responsive defense-related gene expression in tomato (135–137). The cell-free culture filtrate medium of the fungal pathogens *Paecilomyces tenuipes* and *Beauveria bassiana* are also able to induce ET-responsive defense-related gene expression in tomato roots and suppress bacterial wilt disease (Takahashi et al., unpublished results). Hence, insect pathogen-mediated activation of plant defense system to pathogens seems to be consistent with a potential role of insect pathogens to protect plants against the attack of plant pathogens in nature, which has been proposed as the “Bodyguard hypothesis” (138).

Since activation of PTI in plants by recognizing MAMPs of non-pathogenic microorganisms seems to be widely distributed phenomenon (16), PTI would contribute to reduce disease incidence through the defense system activated by environmental microorganisms in nature. In recent years, research on harnessing beneficial functions of members of the plant microbiome to make them useful in sustainable crop protection emerged as one of the frontiers in plant science research (103, 139–141). Besides MAMPs, many other microbe-associated small molecules have been identified as being important for beneficial host-microbe interactions (118), providing useful tools for sustainable protection of future crops.

## Perspective of healthy growth strategy by beneficial microbes in animals and plants

**Figure 6:**
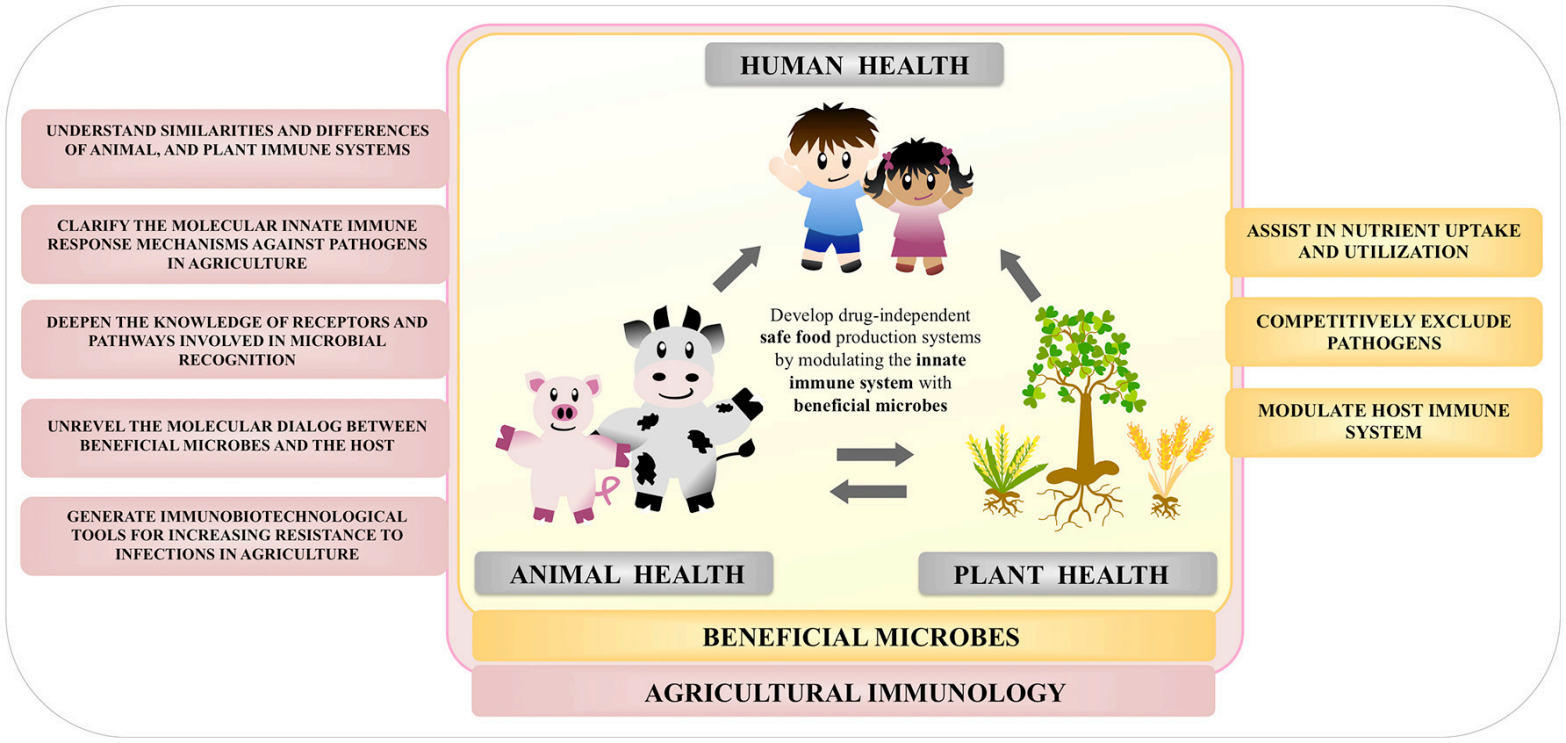
Overview of the importance of the study of beneficial microbes within the field of agricultural immunology.

Modern animal and crop production practices are associated with the regular use of antimicrobials, potentially increasing selection pressure on bacteria to become resistant. Considering the global intention of organizations to significantly reduce the use of antimicrobials in agriculture, the need for novel strategies to improve resistance of animal and plants against pathogens became a top priority. Agricultural Immunology is a developing research field that fuses animal, marine, and plant immunology, all of which have been previously regarded as separate topics. Agricultural Immunology therefore aims to plant the seeds for developing drug-independent safe food production systems by modulating animal and plant innate immune system (Figure 6).

Deciphering animal–microbe and plant–microbe interactions is a promising aspect to understand the benefits and the pathogenic effect of microbes in the agricultural field. The advancement in sequencing technologies and various “omics” tool has impressively accelerated the research in biological sciences in this area. The development of new techniques in the post–genomic era has greatly enhanced our understanding of the regulation of animal and plant defense mechanisms against pathogens, and also their interaction with beneficial microbes. Thus, animal–microbe and plant–microbe associations can now be studied at a speed and depth as never before. However, a major gap in our knowledge is how recognition of beneficial microbes at the gut or root-soil interface drives the whole animal or plant body toward enhanced growth and elevated stress resistance. The first steps toward unraveling the molecular dialog between hosts and beneficial microbes eliciting distal immunological effects have been made, but major questions still need to be resolved.

The aim of the “agricultural immunobiotic approach” is to repair the deficiencies in the microbiota and restore the host's resistance to disease through the use of beneficial immunomodulatory microorganisms. Such treatments do not introduce any foreign chemicals into the animal gut or plant root and does not run the risk of contaminating and introducing hazardous chemicals into the food chain. We hope to convey the enthusiasm of this rapidly advancing field as an area of active basic and applied research that is at the cusp of exploitation to address pressing plant and animal health problems worldwide.

## Author contributions

All authors listed have made a substantial, direct and intellectual contribution to the work, and approved it for publication.

### Conflict of interest statement

The authors declare that the research was conducted in the absence of any commercial or financial relationships that could be construed as a potential conflict of interest. The reviewer TM and handling editor declared their shared affiliation.
